# Pyronaridine–artesunate and artemether–lumefantrine for the treatment of uncomplicated *Plasmodium falciparum* malaria in Kenyan children: a randomized controlled non-inferiority trial

**DOI:** 10.1186/s12936-018-2340-3

**Published:** 2018-05-15

**Authors:** Johanna M. Roth, Patrick Sawa, Nicodemus Makio, George Omweri, Victor Osoti, Selpha Okach, Felix Choy, Henk D. F. H. Schallig, Pètra Mens

**Affiliations:** 1 Department of Medical Microbiology, Laboratory for Clinical Parasitology, Academic Medical Center, Meibergdreef 9, 1105 AZ Amsterdam, The Netherlands; 2 Human Health Division, International Centre of Insect Physiology and Ecology, Mbita Point, Kenya

**Keywords:** *Plasmodium falciparum*, Malaria, Paediatric, Pyronaridine–artesunate, Artemether–lumefantrine, Kenya

## Abstract

**Background:**

Pyronaridine–artesunate is a novel artemisinin-based combination therapy. The efficacy and safety of pyronaridine–artesunate were compared with artemether–lumefantrine for the treatment of uncomplicated *Plasmodium falciparum* malaria in children.

**Methods:**

This phase III open-label randomized controlled non-inferiority trial was conducted in Western Kenya. Children aged 6 months to ≤ 12 years with a bodyweight > 5 kg and microscopically confirmed *P. falciparum* malaria were randomly assigned in a 1:1 ratio to orally receive pyronaridine–artesunate or artemether–lumefantrine, dosed according to bodyweight, for 3 days.

**Results:**

Of 197 participants, 101 received pyronaridine–artesunate and 96 received artemether–lumefantrine. The day-28 adequate clinical and parasitological response in the per-protocol population, PCR-corrected for reinfections, was 98.9% (93/94, 95% CI 94.2–99.8) for pyronaridine–artesunate and 96.4% (81/84, 95% CI 90.0–98.8) for artemether–lumefantrine. Pyronaridine–artesunate was found to be non-inferior to artemether–lumefantrine: the treatment difference was 2.5% (95% CI − 2.8 to 9.0). Adverse events occurred in 41.6% (42/101) and 34.4% (33/96) of patients in the pyronaridine–artesunate group and the artemether–lumefantrine group, respectively. No participants were found to have alanine or aspartate aminotransferase levels > 3 times the upper limit of normal.

**Conclusions:**

Pyronaridine–artesunate was well tolerated, efficacious and non-inferior to artemether–lumefantrine for the treatment of uncomplicated *P. falciparum* malaria in Kenyan children. Results are in line with previous reports and inclusion of pyronaridine–artesunate in paediatric malaria treatment programmes should be considered.

This study is registered at clinicaltrials.gov under NCT02411994. Registration date: 8 April 2015. https://clinicaltrials.gov/ct2/show/NCT02411994?term=pyronaridine–artesunate&cond=Malaria&cntry=KE&rank=1

**Electronic supplementary material:**

The online version of this article (10.1186/s12936-018-2340-3) contains supplementary material, which is available to authorized users.

## Background

*Plasmodium falciparum* malaria still has high morbidity and mortality rates. The World Health Organization (WHO) estimated that 216 million malaria cases and 445,000 deaths, mainly due to *P. falciparum*, occurred worldwide in 2016 [1]. Most endemic countries have adopted artemisinin-based combination therapy (ACT) as the first-line treatment for *P. falciparum* malaria, as recommended by the WHO [2]. However, resistance against commonly used ACT medicines is rising in South-East Asia and the potential spread to African countries is a major concern [3, 4].

New drugs are under development that offer possible alternatives to currently used ACT medicines. One of these alternatives is pyronaridine–artesunate (PA), which is a fixed-dose combination therapy developed by Shin Poong Pharmaceuticals (South Korea), in partnership with Medicines for Malaria Venture (MMV) [5]. PA received a positive scientific opinion from the European Medicines Agency (EMA) [6] and has some advantages over the frequently used artemether–lumefantrine combination (AL): it does not require fatty food for optimal absorption, it needs to be taken only once per day (instead of twice) and the longer half-life (13.2 days for pyronaridine compared to 3.2 days for lumefantrine [7, 8]) may prevent early reinfection.

In phase III studies, PA was found to be well tolerated and efficacious for the treatment of uncomplicated *P. falciparum* malaria and the blood stage of *Plasmodium vivax* malaria [9–15]. Polymerase chain reaction (PCR)-corrected cure rates were > 95% on day 28 in the per-protocol populations of these studies and > 93% on day 42 [9, 10, 14, 15]. Safety and efficacy were maintained after retreatment of multiple malaria episodes [15]. However, in a recent study in Western Cambodia, in an area known for high prevalence of artemisinin resistance, cure rates just below the 90% WHO-recommended efficacy threshold were found at day 42 [2, 16]. The reason for this lower efficacy remains to be investigated, as the study area knows very limited use of pyronaridine and cross-resistance between partner drugs seems unlikely according to a WHO meeting report of the Technical Expert Group on Drug Efficacy and Response [17].

One study specifically included children ≤ 12 years of age [14], but most participants in other studies evaluating the efficacy and safety of PA were adults and older children, who are expected to have acquired some antimalarial immunity [10, 15]. This immunity could improve treatment outcomes, especially for partly effective drugs [18, 19]. As young children are most vulnerable to malaria and adverse events may be more serious in this patient group [20], it is important to pay special attention to the efficacy and safety of PA in children [15, 21].

Malaria management in children improves with the use of paediatric formulations [22]. Child-friendly soluble PA granules were developed to facilitate easier swallowing, in addition to the tablet formulation for children ≥ 20 kg and adults. Pharmacokinetics were shown to be similar between the PA tablet and granule formulation [23]. Non-inferiority of PA granules to AL was demonstrated in a phase III study evaluating the treatment efficacy for uncomplicated *P. falciparum* malaria in children ≤ 12 years of age from various endemic settings [14], and confirmed by (a subgroup of) Sagara et al. [15]. However, to make future implementation decisions, more studies evaluating the efficacy and safety of PA in children are warranted [13].

In this phase III trial, the efficacy and safety of PA was compared with AL for the treatment of uncomplicated *P. falciparum* malaria in Kenyan children aged ≤ 12 years. The primary objective was to test non-inferiority of PA to AL based on an adequate clinical and parasitological response (ACPR) on day 28, PCR-corrected for reinfections.

## Methods

### Ethics statement

This randomized controlled non-inferiority trial was conducted at St. Jude’s Clinic, Mbita, Western Kenya from October 2015 to June 2016 and from January to August 2017, in accordance with Good Clinical Practice, regulatory requirements and the Declaration of Helsinki (2013). Ethical approval was obtained from the Ethical Review Committee of the Kenya Medical Research Institute (KEMRI) (NON-SSC no. 479) and the Expert Committee on Clinical Trials of the Kenyan Pharmacy and Poisons Board (PPB). The trial protocol was registered at clinicaltrials.gov under NCT02411994. The study had a Data and Safety Monitoring Board (DSMB). Written informed consent from a parent or legal guardian was required for participation; assent was sought from children able to understand the study.

### Patients

Children were eligible to participate if they were 6 months to 12 years of age, weighed at least 5 kg, lived within 10 km from the study clinic and had microscopically confirmed *P. falciparum* mono-infection with 1000–200,000 asexual parasites/µL. Exclusion criteria were complicated or severe malaria, non-*P. falciparum* or mixed *Plasmodium* infection, a history of hepatic and/or renal impairment, any clinically significant illness other than malaria, anaemia with a haemoglobin (Hb) concentration < 6 g/dL, severe malnutrition, treatment with anti-malarial therapy in the previous 2 weeks, known hypersensitivity to artemisinins, previous participation in this study, current participation in other anti-malarial drug intervention studies or not being available for follow-up.

### Randomization and masking

Participants were randomized 1:1 to PA or AL using a computer-generated randomization schedule, provided by the sponsor. The code linking to the treatment was kept in sequentially numbered sealed opaque envelopes. Participants were allocated in order of enrollment to the treatment in the next available envelope. Drugs were administered by pharmacy personnel aware of group assignments. Clinical and parasitological assessments were performed by study staff members blinded to treatment allocation (until completion of data analysis). The sponsor remained blinded to treatment allocation as well.

### Treatment

Study drugs were given orally for 3 days (0, 1 and 2) and were dosed according to body weight (Additional file 1). PA (Shin Poong Pharmaceutical Company, Seoul, South-Korea) was given once daily, directly observed at the study clinic. The tablet form (for children ≥ 20 kg) contained 180 mg pyronaridine–tetraphosphate and 60 mg artesunate per tablet. Granules (for children < 20 kg) were provided in sachets and contained 60 mg pyronaridine–tetraphosphate and 20 mg artesunate per sachet. Oral suspensions were prepared immediately before dosing, whereby granules were stirred into 50 mL lemonade. Residual drug was given by adding 100 mL of water or lemonade to the dosing cup. AL (Novartis, Basel, Switzerland) contained 20 mg artemether and 120 mg lumefantrine per tablet and was taken twice daily. AL was provided in tablet formulation, crushed and prepared like PA granules for children unable to swallow the tablets. The morning dose of AL was administered directly observed at the study clinic. The evening dose was given to the parent/guardian to administer at home. On every day following AL treatment, pharmacy personnel asked the parent or guardian whether study drugs were administered in the evening. All participants received their drugs with food (mandazi—a type of fried bread) or milk, as recommended for AL to optimize absorption. Mandazi was also provided for the AL evening dose. Participants who vomited within 30 min of receiving the first dose were given a repeat dose. Vomiting after repeat dosing or any subsequent dose led to withdrawal from the study and administration of rescue treatment as per local guidelines.

### Procedures

Finger-prick blood samples were collected at screening and on day 0, 1, 2, 3, 7, 14, 28 and 42. Giemsa-stained thick and thin blood smears were prepared according to WHO guidelines [24]. Slides were read by local expert microscopists. A slide was considered negative when 100 high-power fields were examined at 1000× magnification and no parasites were observed. Parasitaemia was determined from thick smears by counting the number of parasites against 200 leukocytes, with the assumption of 8000 leukocytes/µL blood. In case the number of parasites after counting 200 leukocytes was < 100, counting continued up to 500 leukocytes.

Hb was determined on day 0, 3, 7 and 28 by HemoCue (Ängelholm, Sweden). Alanine aminotransferase (ALT) and aspartate aminotransferase (AST) were retrospectively measured on day 3 and 7. If ALT and/or AST levels were > 3× the upper limit of normal (ULN) on day 3 and/or day 7, ALT and AST were measured for day 0 and 28 as well. Bilirubin was also measured in these particular cases. If bilirubin levels were > 2× ULN, alkaline phosphatase (ALP) was measured in order to identify potential Hy’s law cases. Due to logistic constraints, ALT and AST were only measured for the first 150 participants (see "Results" section).

To distinguish between reinfections and recrudescences after parasite reappearance, nested PCR-based genotyping was performed at the Academic Medical Center (Amsterdam, The Netherlands). Using the *msp*1, *msp*2 and *glurp* genes, a recrudescence was defined as at least one matching allelic band in all markers between the baseline sample and the sample at the day of reappearance [25].

### Outcomes

Efficacy outcomes were based on WHO definitions [26]. The primary efficacy outcome was adequate clinical and parasitological response (ACPR) on day 28, corrected for reinfection by genotyping. Secondary efficacy outcomes were ACPR on day 28 without correction for reinfection, ACPR on day 42 with and without correction by genotyping, recrudescence and reinfection rates over 42 days, parasite clearance time (defined as time from first dose to a parasitaemia, determined by two consecutive negative readings 7–25 h apart), fever clearance time (defined as time from first dose to apyrexia, determined by two consecutive normal readings 7–25 h apart), and the proportion of patients with parasite or fever clearance on day 1, 2 and 3. Safety outcomes were incidence of (serious) adverse events, severe anaemia (Hb < 6 g/dL) and the occurrence of hepatotoxicity events (defined as ALT and/or AST > 3× the ULN on day 3 and/or 7).

### Sample size

Assuming a day-28 ACPR of 95% for both treatment regimens, 201 children per intervention arm (402 in total) would provide 91% power to demonstrate non-inferiority of PA compared to AL, with a non-inferiority margin of 7%. After correcting for 10% drop-out, target recruitment was 447 participants. Unfortunately, target recruitment was not reached (see results section) and 197 participants were included, which resulted in a reduction of power to 62% to demonstrate non-inferiority of PA to AL with a non-inferiority margin of 7%.

### Statistical analysis

The intention-to-treat population consisted of all randomized participants who received any amount of study medication, and was the same as the safety population. The per-protocol population included participants who received a full course of study medication, had a known day-28 primary efficacy endpoint and had no major protocol violation. The primary efficacy outcome was evaluated in the per-protocol population. Non-inferiority was demonstrated if the lower limit of the 2-sided 95% confidence interval (CI) for the difference in day-28 cure rate was greater than − 7%. This confidence interval was calculated using the Newcombe-Wilson method without continuity correction. Similar analyses were performed for uncorrected ACPR on day 28, corrected and uncorrected ACPR on day 42 and for the intention to-treat-population. Additionally, Kaplan–Meier analyses were used to compare recrudescence and reinfection rates between intervention groups (log-rank test). Participants without the event (recrudescence or reinfection) or with major protocol deviations were censored at the last available parasite assessment date (before the deviation). Parasite and fever clearance times were also evaluated using Kaplan–Meier estimates and compared between groups (log-rank test). Here, all participants without confirmed clearance on day 3 were censored. Statistical analyses were performed in Stata (version 14.2, Stata Corporation, Texas, USA).

## Results

### Participants

**Fig. 1:**
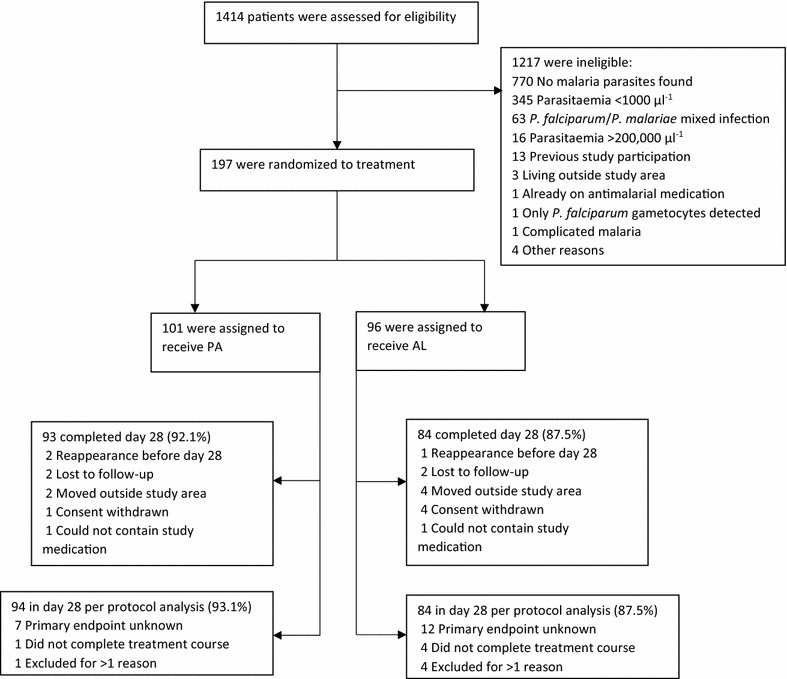
Participant flow. Number of patients screened, randomized and included in the per-protocol population. Some patients had more than one reason for exclusion from the per-protocol population

A total of 197 patients were included and randomized, 101 to PA and 96 to AL (Fig. 1). Of the 101 participants in the PA-group, 60 received tablets and 41 received granules. Less participants were included than initially planned. Most likely explanations were exceptional drought during the study period, many low-density infections and more mixed infections than expected [9.8% (63/644) of all microscopy positive patients at screening had a *P. falciparum/Plasmodium malariae* mixed infection]. All participants received at least one dose of study medication and were included in the intention-to-treat analysis. Most patients (89.8%) completed day 28. Slightly more patients were excluded from the per-protocol population in the AL group (12.5%) compared to the PA group (6.93%), because more participants in the AL group moved away or withdrew consent (Fig. 1). The most common reason for exclusion from the per-protocol population was missing data for the primary outcome. Baseline characteristics were similar between intervention groups (Table 1).

**Table 1:** Baseline characteristics of study participants at enrolment

	Pyronaridine–artesunate (n = 101)	Artemether–lumefantrine (n = 96)
Male sex, n (%)	53 (52.5)	48 (50.0)
Mean age, years (SD) [range]	6.9 (2.9) [1.6–12]	6.4 (3.3) [0.9–12]
Age category, years, n (%)		
≤ 1	0	2 (2.1)
> 1 to < 5	31 (30.7)	31 (32.3)
5–12	70 (69.3)	63 (65.6)
Mean weight, kg (SD) [range]	23.0 (8.4) [10–44]	22.4 (9.2) [8.5–45]
Mean hemoglobin, g/dL (SD) [range]	11.8 (1.9) [6.2–15.8]	11.9 (2.1) [6.8–16.4]
Mean temperature, °C (SD) [range]	37.5 (1.2) [35.1–39.8]	37.3 (1.2) [35.2–39.9]
Fever (temperature > 37.5 °C), n (%)	53 (52.5)	40 (41.7)
Geometric mean asexual parasitaemia, µL^−1^ (95% CI)	24,420.6 (18,837.6–31,658.3)	23,672.5 (18,576.2–30,167.0)
Gametocyte prevalence-microscopy, n (%)	2 (1.98)	4 (4.17)

### Efficacy

PCR-corrected ACPR on day 28 in the per-protocol population, the primary efficacy outcome, was 98.9% (95% CI 94.2–99.8) in the PA group and 96.4% (95% CI 90.0–98.8) in the AL group. The treatment difference was 2.5 percentage points (95% CI − 2.8 to 9.0) and non-inferiority of PA compared to AL was demonstrated (Table 2). On day 42, the PCR-corrected ACPR in the per-protocol population was 94.8% (95% CI 87.4–98.0) in the PA group and 91.0% (95% CI 81.8–95.8) in the AL group, with a treatment difference of 3.8 (95% CI − 5.1 to 13.5). The uncorrected ACPR on day 28 was 91.6% (95% CI 84.3–95.7) in the PA group and 88.2% (95% CI 79.7–93.5) in the AL group. On day 42, the uncorrected ACPR was 77.9 (95% CI 68.1–85.4) in the PA group and 72.7 (95% CI 61.9–81.4) in the AL group.

**Table 2:** Adequate clinical and parasitological response (ACPR) in the per-protocol population

	Pyronaridine–artesunate	Artemether–lumefantrine	Difference (95% CI)
Day 28			
PCR-corrected ACPR, n/N^b^	93/94	81/84	
% (95% CI)	98.9 (94.2 to 99.8)	96.4 (90.0 to 98.8)	2.5 (− 2.8 to 9.0)^a^
Total no. of failures	1 (1.1)	3 (3.6)	
No. with early treatment failure	0	0	
No. with late clinical failure	0	0	
No. with late parasitological failure	1 (1.1)	3^c^ (3.6)	
No. with missing data	7 (7.4)	12 (14.3)	
Uncorrected ACPR, n/N	87/95	75/85	
% (95% CI)	91.6 (84.3 to 95.7)	88.2 (79.7 to 93.5)	3.3 (− 5.7 to 12.8)
Total no. of failures	8 (8.4)	10 (11.8)	
No. with early treatment failure	0	0	
No. with late clinical failure	1 (1.1)	1 (1.2)	
No. with late parasitological failure	7 (7.4)	9 (10.6)	
No. with missing data	6 (6.4)	11 (12.9)	
Day 42			
PCR-corrected ACPR, n/N^b^	73/77	61/67	
% (95% CI)	94.8 (87.4 to 98.0)	91.0 (81.8 to 95.8)	3.8 (− 5.1 to 13.5)
Total no. of failures	4 (5.2)	6 (9.0)	
No. with early treatment failure	0	0	
No. with late clinical failure	1 (1.3)	0	
No. with late parasitological failure	3^c^ (3.9)	6^d^ (9.0)	
No. with missing data	24 (31.2)	29 (43.3)	
Uncorrected ACPR, n/N	67/86	56/77	
% (95% CI)	77.9 (68.1 to 85.4)	72.7 (61.9 to 81.4)	5.2 (− 8.0 to 18.4)
Total no. of failures	19 (22.1)	21 (27.3)	
No. with early treatment failure	0	0	
No. with late clinical failure	5 (5.8)	2 (2.6)	
No. with late parasitological failure	14 (16.3)	19 (24.7)	
No. with missing data	15 (17.4)	19 (24.7)	

Data are n (%) unless otherwise indicated

Participants with a reinfection before day 28 were included in the day-28 per-protocol population for the uncorrected analysis. In the PCR-corrected analysis, however, patients with a reinfection before day 28 were excluded from the analysis because data were missing on day 28 (and there was no established recrudescence). On day 42, the per-protocol population was defined similarly

^a^Non-inferiority of pyronaridine–artesunate to artemether–lumefantrine is demonstrated if the lower limit of the 95% confidence interval (CI) of the difference in ACPR is > − 7%

^b^PCR-corrected for reinfection by *msp1*, *msp2* and *glurp* genotyping

^c^One of the late parasitological failures had indeterminate genotyping and was marked as recrudescence

^d^Two of the late parasitological failures had indeterminate genotyping and were marked as recrudescence

In the intention-to-treat population, day-28 PCR-corrected ACPR was 92.1% (95% CI 85.1–95.9) in the PA group and 84.4% (95% CI 75.8–90.3) in the AL group. Day-42 PCR-corrected ACPR was 72.3% (95% CI 62.9–80.1) in the PA group and 63.5% (95% CI 53.6–72.5) in the AL group. All outcomes for the intention-to-treat population are shown in Table 3.

**Table 3:** Adequate clinical and Parasitological response (ACPR) in the intention-to-treat population

	Pyronaridine–artesunate (n = 101)	Artemether–lumefantrine (n = 96)	Difference (95% CI)
Day 28			
PCR-corrected ACPR, n/N^a^	93/101	81/96	
% (95% CI)	92.1 (85.1 to 95.9)	84.4 (75.8 to 90.3)	7.7 (− 1.4 to 17.1)
Total no. of failures	8 (7.9)	15 (15.6)	
No. with missing data	6 (5.9)	11 (11.5)	
No. with early treatment failure	0	0	
No. with late clinical failure	0	0	
No. with late parasitological failure	1 (1.0)	3^b^ (3.1)	
No. with reinfection < day 28	1 (1.0)	1 (1.0)	
Uncorrected ACPR, n/N	87/101	75/96	
% (95% CI)	86.1 (78.1 to 91.6)	78.1 (68.9 to 85.2)	8.0 (− 2.7 to 18.8)
Total no. of failures	14 (13.9)	21 (21.9)	
No. with missing data	6 (5.9)	11 (11.5)	
No. with early treatment failure	0	0	
No. with late clinical failure	1 (1.0)	1 (1.0)	
No. with late parasitological failure	7 (6.9)	9 (9.4)	
Day 42			
PCR-corrected ACPR, n/N^a^	73/101	61/96	
% (95% CI)	72.3 (62.9 to 80.1)	63.5 (53.6 to 72.5)	8.7 (− 4.3 to 21.4)
Total no. of failures	28 (27.7)	35 (36.5)	
No. with missing data	15 (14.9)	19 (19.8)	
No. with early treatment failure	0	0	
No. with late clinical failure	1 (1.0)	0	
No. with late parasitological failure	3^b^ (3.0)	6^c^ (6.3)	
No. with reinfection < day 42	9 (8.9)	10 (10.4)	
Uncorrected ACPR, n/N	67/101	56/96	
% (95% CI)	66.3 (56.7 to 74.8)	58.3 (48.3 to 67.7)	8.0 (− 5.5 to 21.1)
Total no. of failures	34 (33.7)	40 (41.7)	
No. with missing data	15 (14.9)	19 (19.8)	
No. with early treatment failure	0	0	
No. with late clinical failure	5 (5.0)	2 (2.1)	
No. with late parasitological failure	14 (13.9)	19 (19.8)	

Data are n (%) unless otherwise indicated

^a^PCR-corrected for reinfection by *msp1*, *msp2* and *glurp* genotyping

^b^One of the late parasitological failures had indeterminate genotyping and was marked as recrudescence

^c^Two of the late parasitological failures had indeterminate genotyping and were marked as recrudescence

**Fig. 2:**
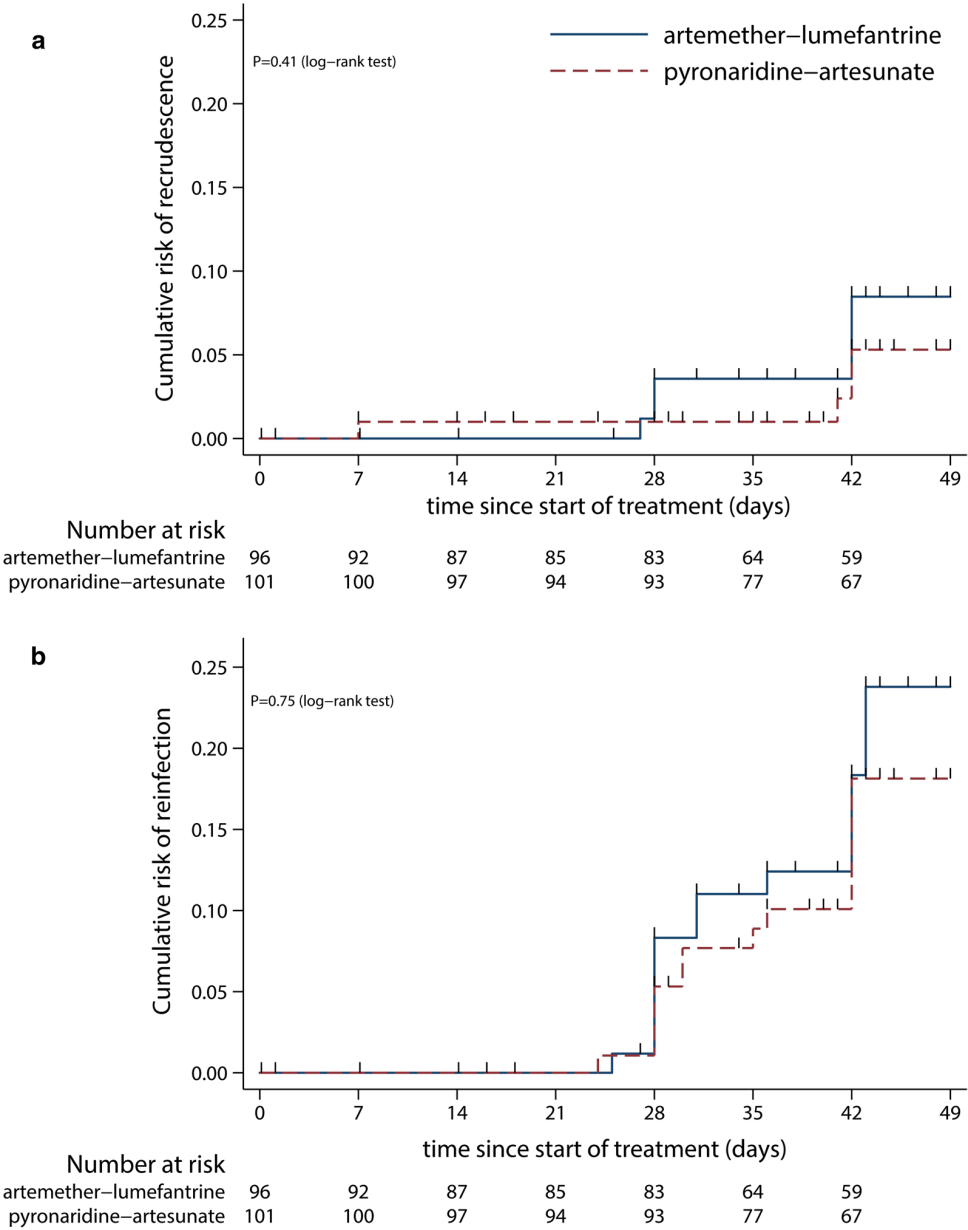
Kaplan–Meier estimates for **a** rate of recrudescence and **b** rate of reinfection (in the intention-to-treat population). Black markers represent censored cases. Participants without the event (recrudescence or reinfection) were censored at the last available parasite assessment date

Kaplan–Meier estimates of recrudescence in the intention-to-treat population were 5.31% (95% CI 2.00–13.7) in the PA group and 8.47% (95% CI 3.85–18.1) in the AL group (through day 42). Estimates of reinfection were 18.1% (95% CI 11.3–28.4) and 23.8% (95% CI 13.4–40.1) for PA and AL, respectively. No difference in recrudescence (P = 0.41, log-rank test) or reinfection (P = 0.75, log-rank test) rates was found between the study groups (Fig. 2).

**Fig. 3:**
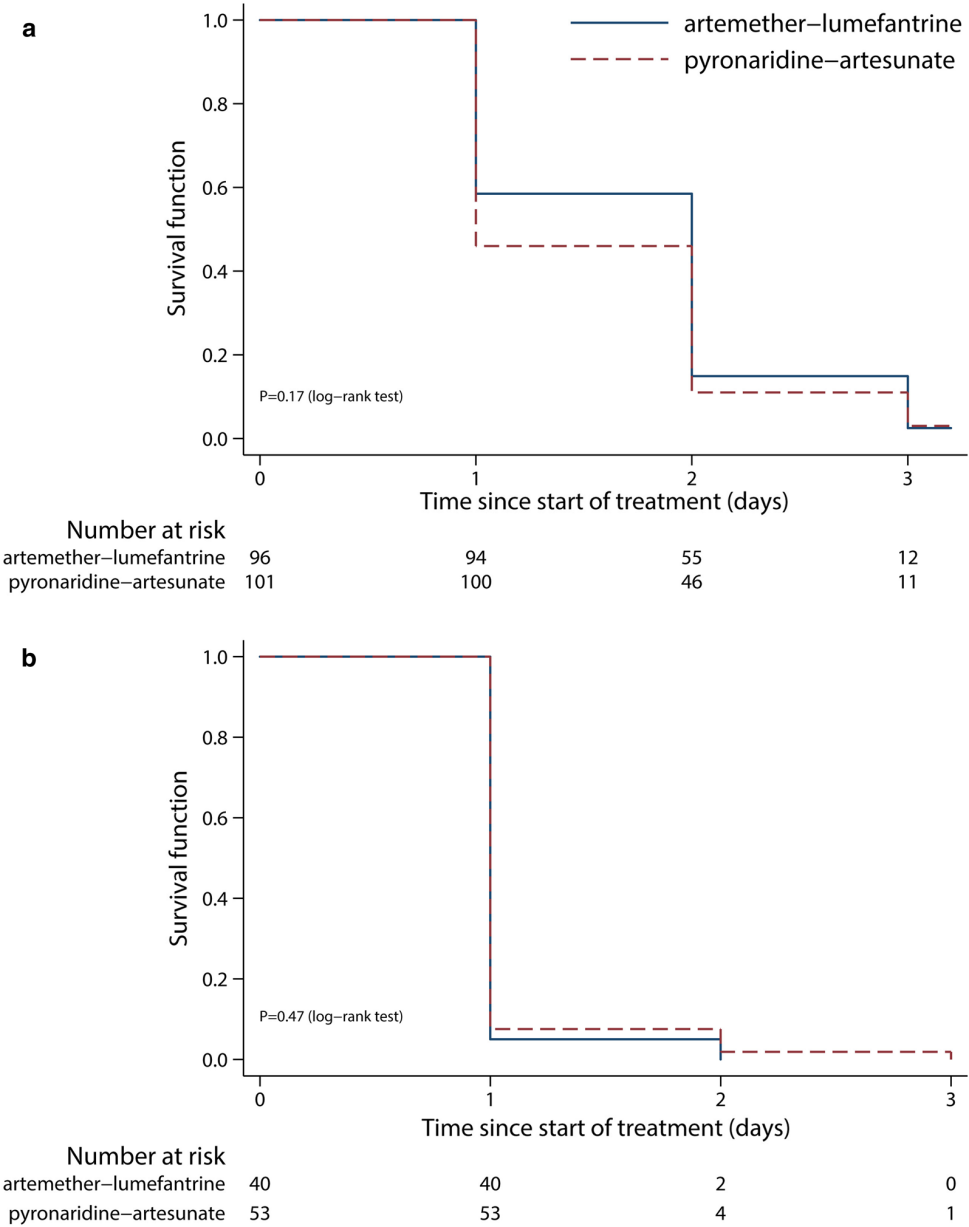
Kaplan–Meier estimates for **a** time to parasite clearance (intention-to-treat population) and **b** time to fever clearance. Participants without the event (parasite or fever clearance) were censored at the last available parasite or fever assessment date

Kaplan–Meier estimates of parasite clearance at day 3 were 97.0% (95% CI 92.2–99.2) in the PA group and 97.5% (95% CI 92.3–99.5) in the AL group. There was no difference between PA and AL in the parasite clearance time (P = 0.17, log-rank test) (Fig. 3a). Median time to parasite clearance and the proportion of patients with clearance on day 1, 2 and 3 are presented in Additional file 2.

All patients were fever-free on day 3. Fever clearance time was similar between intervention groups (P = 0.47, log-rank test) (Fig. 3b). Median time to fever clearance and the proportion of patients with fever clearance on day 1, 2 and 3 are presented in Additional file 2.

At baseline, gametocyte prevalence by microscopy was 1.98% (95% CI 0.54–6.93) for PA and 4.17% (95% CI 1.63–10.2) for AL (Table 1). On day 7, 3.00% of participants in the PA group (95% CI 1.03–8.45) and 1.09% (95% CI 0.19–5.91) of participants in the AL group had microscopically detectable gametocytes.

### Safety

Adverse events occurred in 41.6% of patients in the PA group and 34.4% of patients in the AL group (Table 4). One patient in the PA group was excluded on day 0 and one patient in the AL group was excluded on day 1, due to repeated vomiting. There were no serious adverse events observed during the study.

**Table 4:** Summary of adverse events in the intention-to-treat population

	Pyronaridine–artesunate (n = 101)	Artemether–lumefantrine (n = 96)
Adverse event leading to discontinuation of study drug		
Vomiting	1 (0.99)	1 (1.04)
Adverse event of any cause		
Patients with at least 1 event	42 (41.6)	33 (34.4)
Headache	10 (9.90)	10 (10.4)
Vomiting	10 (9.90)	5 (5.21)
Cough	18 (17.8)	15 (15.6)
Abdominal pain	3 (2.97)	6 (6.25)
Anorexia	4 (3.96)	6 (6.25)
Diarrhoea	2 (1.98)	4 (4.17)
Chills	2 (1.98)	0
Fatigue	4 (3.96)	1 (1.04)
Myalgia	1 (0.99)	1 (1.04)
Nasopharyngitis	3 (2.97)	4 (4.17)
Dizziness	1 (0.99)	0
Skin rash	2 (1.98)	1 (1.04)
Dark urine	1 (0.99)	1 (1.04)
Ear pain	0	1 (1.04)
Chest pain	0	2 (2.08)
Throat pain	1 (0.99)	0
Neck pain	0	1 (1.04)

Data are n (%)

No patients had instances of post-baseline ALT or AST levels > 3 times the ULN. No potential Hy’s law cases were identified. Mean ALT levels on day 3 were 11.0 U/L (n = 66, SD: 6.3) in the PA group and 11.8 U/L (n = 61, SD: 8.6) in the AL group. On day 7, mean ALT levels were 11.7 U/L (n = 65, SD: 7.5) in the PA group and 11.2 U/L (n = 60, SD: 6.0) in the AL group. Mean AST levels on day 3 were 19.6 U/L (n = 62, SD: 7.6) in the PA group and 21.2 U/L (n = 52, SD: 8.9) in the AL group. On day 7, mean AST levels were 21.0 U/L (n = 62, SD: 8.0) in the PA group and 21.5 U/L (n = 63, SD: 8.2) in the AL group (Additional file 3).

The lowest Hb value measured was 6.2 g/dL. Similar Hb changes from baseline were found in the two intervention groups. Mean Hb concentrations on day 3 compared to baseline decreased 0.8 and 0.6 g/dL for the PA and the AL group, respectively, and recovered by day 28 (Additional file 3).

## Discussion

Treatment of uncomplicated *P. falciparum* malaria in Kenyan children aged ≤ 12 years with PA tablets or granules resulted in a PCR-corrected ACPR of 98.9% on day 28 in the per-protocol population, which is above the 95% standard set by the WHO in 2010 for the selection of a new or alternative anti-malarial [27]. However, the newest 2015 WHO guidelines for the treatment of malaria do not specifically state this 95% threshold [2]. PCR-corrected ACPR in the AL-group was 96.4% and non-inferiority of PA to AL was demonstrated. These and day 42 PCR-corrected efficacy estimates were consistent with previously reported ACPR rates in adults and children with *P. falciparum* malaria living in areas without artemisinin resistance [9, 10, 14].

The ACPR estimates in the uncorrected analysis, however, were lower compared to reports from Tshefu et al. [10] and Rueangweerayut et al. [9], but very similar to those presented reported by Kayentao et al. [14], due to a higher number of reinfections. This may be explained by differences in transmission rates between the studies or by age differences (mean age 6.9 and 4.9 years in the current study and Kayentao et al. [14], respectively, *versus* 17.2 and 25 years in the other studies [9, 10]). Better immunity to *P. falciparum* in the older population might have contributed to a longer prophylactic effect [14].

No difference in the rate of recrudescence was found between study groups. Reinfections were anticipated to occur later in the PA group compared to the AL group, as observed in a previous trial, due to the longer half-life of pyronaridine [10]. However, no differences in reinfection rates were found between treatment groups, similar to the findings of Kayentao et al. [14]. This disagreement may be related to differences in the patient population or, in the present study, to the sample size.

Both treatments rapidly cleared *P. falciparum* and no difference in parasite clearance time was observed between the two study groups, in contrast to previous trials where parasite clearance time was faster with PA [9, 10, 14]. All patients were fever free on day 3 and no difference in fever clearance time was observed, in line with a previous study [10], while another trial showed faster fever clearance with PA [14].

Safety findings were similar to previous reports on PA and pyronaridine and artesunate monotherapy [9–11, 14, 15, 23, 28–30]. Importantly and in contrast to former studies, none of the ALT and/or AST concentrations measured was > 3 times the ULN on day 3 and/or 7. No indication of hepatotoxicity was found.

A limitation of this study is that the initially planned sample size was not reached, which led to a reduction of power. However, PA efficacy estimates were never lower than AL estimates, and for the primary efficacy endpoint the lower limit of the 95% CI was − 2.8, whereas − 7 was the limit for demonstrating non-inferiority (Table 2). This strengthens this studies’ conclusion that PA was non-inferior to AL. The present study included only 2 children ≤ 1 year in the AL group (both had an ACPR at day 28), and more efficacy and safety data on this patient group is still needed for PA [15]. Furthermore, the study was conducted from an outpatient clinic and participants were not hospitalized. It was, therefore, logistically not feasible to collect more than one sample per day, while other studies collected a sample every 8 h for the first 3 days. The latter provides a more detailed insight into parasite and fever clearance dynamics. Concerning hepatotoxicity, ALT and AST were not measured for all participants, due to logistic constraints and because not enough serum was available for all participants. The hepatic enzyme profile is, therefore, not complete.

## Conclusions

This study adds much needed data to the efficacy and safety profile of PA in children. PA granules and tablets were shown to be effective and well tolerated for the treatment of uncomplicated *P. falciparum* malaria in Kenyan children aged ≤ 12 years. These findings are in line with previous reports and inclusion of pyronaridine–artesunate in paediatric malaria treatment programs should be considered.

## Additional files

**Additional file 1.** Weight-based dosing of pyronaridine–artesunate and artemether–lumefantrine.

**Additional file 2.** Parasite and fever clearance at day 1, 2 and 3.

**Additional file 3.** Haemoglobin (Hb), alanine aminotransferase (ALT) and aspartate aminotransferase (AST) values.
