# Supplementary material for: Pyronaridine–artesunate and artemether–lumefantrine for the treatment of uncomplicated Plasmodium falciparum malaria in Kenyan children: a randomized controlled non-inferiority trial
Source: Malar J. 2018 May 15;17:199. doi: 10.1186/s12936-018-2340-3 (PMC5952621; doi:10.1186/s12936-018-2340-3)
Supplement: Supplementary file 2 — Additional file 2. Parasite and fever clearance at day 1, 2 and 3. [file 12936_2018_2340_MOESM2_ESM.pdf]

Additional file 2: Parasite and fever clearance time

	**pyronaridine-artesunate**	**artemether-lumefantrine**
**Parasite clearance[a]**		
Asexual parasite clearance on day 3, n/N (%)	97/101 (96.0)	91/96 (94.8)
Median clearance time, days (95% CI)	1 (1-2)	2 (1-2)
Participants with clearance, % (95% CI) at:		
Day 1	55.5 (45.7-64.8)	41.7 (32.3-51.7)
Day 2	89.1 (81.5-93.8)	84.4 (75.8-90.3)
Day 3	96.0 (90.3-98.5)	94.8 (88.4-97.8)
**Fever clearance**		
Fever clearance on day 3, n/N (%)	53/53 (100)	40/40 (100)
Median clearance time, days (95% CI)	1 (1-1)	1 (1-1)
Participants with clearance, % (95% CI) at:		
Day 1	94.3 (84.6-98.1)	95.0 (83.5-98.6)
Day 2	98.1 (90.1-99.7)	100 (91.2-100)
Day 3	100 (93.2-100)	100 (91.2-100)

[a] Patients with missing data, for whom parasite clearance could not be determined, were treated as not having cleared parasites on day 3 (1 in the PA group and 3 in the AL group).
