# Supplementary material for: Pyronaridine–artesunate and artemether–lumefantrine for the treatment of uncomplicated Plasmodium falciparum malaria in Kenyan children: a randomized controlled non-inferiority trial
Source: Malar J. 2018 May 15;17:199. doi: 10.1186/s12936-018-2340-3 (PMC5952621; doi:10.1186/s12936-018-2340-3)
Supplement: Supplementary file 3 — Additional file 3. Haemoglobin (Hb), alanine aminotransferase (ALT) and aspartate aminotransferase (AST) values. [file 12936_2018_2340_MOESM3_ESM.pdf]

Additional file 3: Haemoglobin (Hb), alanine aminotransferase (ALT) and aspartate aminotransferase (AST) values.

	Day	n	pyronaridine-artesunate	n	artemether-lumefantrine
**Hb, g/dL**	**Baseline**	101	11.8 (1.9) [6.2-15.8]	96	11.9 (2.1) [6.8-16.4]
	**3**	97	11.0 (1.8) [6.2-14.8]	89	11.3 (2.0) [6.3-15.6]
	**7**	99	11.4 (1.6) [7.6-14.4]	91	11.6 (1.7) [6.7-14.8]
	**28**	90	12.0 (1.5) [8.2-15.9]	80	12.0 (1.8) [6.9-15.2]
**ALT, U/L**	**3**	66	11.0 (6.3) [0-29]	61	11.8 (8.6) [0-45]
	**7**	65	11.7 (7.5) [0-39]	60	11.2 (6.0) [0-24]
**AST, U/L**	**3**	62	19.6 (7.6) [5-41]	52	21.2 (8.9) [1-46]
	**7**	62	21.0 (8.0) [7-52]	63	21.5 (8.2) [7-49]

Data are mean (SD) [range] or number.
